# Supplementary material for: Content-rich biological network constructed by mining PubMed abstracts
Source: BMC Bioinformatics. 2004 Oct 8;5:147. doi: 10.1186/1471-2105-5-147 (PMC528731; doi:10.1186/1471-2105-5-147)
Supplement: Additional File 5 — The original Chilibot query results of the term "long-term potentiation (LTP)" and 22 other terms, limiting the latest references analyzed to the years 1990, 1995, 2000, and 2004. [file 1471-2105-5-147-S5.bz2 › chilibotAdditionalFile5/ltp2004/html/ZIF268.html]

 


**ZIF268** (Input: ZIF268 ) 

---


**Google Searches:** Entire Web  | EDU domain only  | PDF files only

.

**External Links:** OMIM |
LocusLink
| Swissprot
| GeneCards

  
**Maps of ZIF268**

Simple
Complete
graph in
radiant
tree
square
layout.

**New Hypothesis !**

**Synonyms** 

- zif268   [PubMed]

**Synopsis**

- We show that pharmacological blockade of MAPK ERK phosphorylation, functional inactivation of CREB in an inducible transgenic mouse and inactivation of **zif268** in a mutant mouse result in a similar deficit in long term recognition memory.  Philos Trans R Soc Lond B Biol Sci, 2003 
  [20]

- These results suggest that LTP and spatial training engage a common pathway utilizing an increase in mRNA for raf B, and demonstrate a dissociation between LTP and spatial learning with respect to expression of **zif268** and syntaxin 1B.  Neurosci Lett, 1998 
  [20]

- Together, these results suggest that MAPK, PI 3 kinase  [PI-3K]  and JNK each play a role in the induction of **zif268** gene expression by NGF in PC12D cells.  J Biochem (Tokyo), 1999 
  [20]

- We show that spatial learning is particularly sensitive to reduced levels of **Zif268**, as one half of the complement of **Zif268** in heterozygous mice is insufficient to maintain spatial long term memories.  Hippocampus, 2002 
  [16]

- We show that ERKI II is necessary for the upregulation of the proteins encoded by the immediate early genes **Zif268** and Homer after the induction of LTP in the dentate gyrus by tetanic stimulation of the perforant path in vivo or by BDNF stimulation of primary cortical cultures.  J Neurosci, 2002 
  [16]

- somaticaction potentials, induced by backfiring the cells, were found to be sufficient for phosphorylation of extracellular signal regulated kinase 1 2 and cAMP response element binding protein, as well as for induction of **zif268**.  Proc Natl Acad Sci U S A, 2002 
  [15]

- However, the TrkA isoform containing the variable exon showed significantly higher activation by NT 3, which was detected by stimulation of TrkA autophosphorylation, induction of **ZIF268** transcription, and cellular differentiation.  Proc Natl Acad Sci U S A, 1994 
  [15]

- Non toxic concentrations of anisomycin were found to stimulate these enzyme activities as well as the expression of the early response genes c jun, c fos and **zif268**, and to inhibit NGF induced neurite formation.  Eur J Neurosci, 2000 
  [15]

- From this data we conclude that the synapsin I gene is a target of the **zif268** transcription factor.  J Biol Chem, 1994 
  [14]

- the 50 train stimulus pattern resulted in a robust induction of c fos and c jun mRNA, in addition to **zif268** and junB.  J Neurosci, 1993 
  [14]

- At the age when these animals developed cognitive dysfunction, they had reduced mRNA expression of several genes essential for long term potentiation  [LTP]  and memory formation Arc, **Zif268**, NR2B, GluR1, Homer 1a, Nur77 TR3 .  J Neurosci, 2003 
  [11]

- From these data, we conclude that the **zif268** egr 1 binding site in the synaptobrevin II promoter is NOT functionally active.  Eur J Biochem, 1996 
  [11]

- Furthermore, THC induced phosphorylation of the transcription factor Elk 1, and up regulation of **zif268** mRNA expression are blocked by SL327, a specific inhibitor of MAPK ERK kinase MEK, the upstream kinase of ERK, as well as SCH 23390.  Eur J Neurosci, 2001 
  [11]

- Finally, we investigated the expression of two immediate early genes, activity regulated cytoskeleton associated protein and **Zif268**, both of which are required for generation of late, mRNA synthesis dependent LTP.  J Neurosci, 2002 
  [11]

- Sequencing of the 5 flanking region revealed potential cis acting elements for multiple transcriptional regulatory factors including Sp1, **zif268**, Ets, CREB, and PuF sites.  J Biol Chem, 1997 
  [10]
